# Identification of Potential Key Genes Associated with Adipogenesis through Integrated Analysis of Five Mouse Transcriptome Datasets

**DOI:** 10.3390/ijms19113557

**Published:** 2018-11-12

**Authors:** Song Zhang, Li Wang, Shijun Li, Wenzhen Zhang, Xueyao Ma, Gong Cheng, Wucai Yang, Linsen Zan

**Affiliations:** 1 College of Animal Science and Technology, Northwest A&F University, Yangling 712100, China; zhangsong@nwafu.edu.cn (S.Z.); lw@nwafu.edu.cn (L.W.); lishijun1990cn@nwafu.edu.cn (S.L.); zhangwz@nwafu.edu.cn (W.Z.); maxueyao@nwafu.edu.cn (X.M.); chenggong@nwafu.edu.cn (G.C.); yangwucai111@nwafu.edu.cn (W.Y.); 2 National Beef Cattle Improvement Center, Northwest A&F University, Yangling 712100, China

**Keywords:** gene expression omnibus, adipogenesis, integrated bioinformatics, robust rank aggregation, differentially expressed genes

## Abstract

Adipose tissue is the most important energy metabolism and secretion organ, and these functions are conferred during the adipogenesis process. However, the cause and the molecular events underlying adipogenesis are still unclear. In this study, we performed integrated bioinformatics analyses to identify vital genes involved in adipogenesis and reveal potential molecular mechanisms. Five mouse high-throughput expression profile datasets were downloaded from the Gene Expression Omnibus (GEO) database; these datasets contained 24 samples of 3T3-L1 cells during adipogenesis, including 12 undifferentiated samples and 12 differentiated samples. The five datasets were reanalyzed and integrated to select differentially expressed genes (DEGs) during adipogenesis via the robust rank aggregation (RRA) method. Functional annotation of these DEGs and mining of key genes were then performed. We also verified the expression levels of some potential key genes during adipogenesis. A total of 386 consistent DEGs were identified, with 230 upregulated genes and 156 downregulated genes. Gene Ontology (GO) analysis showed that the biological functions of the DEGs primarily included fat cell differentiation, lipid metabolic processes, and cell adhesion. Kyoto Encyclopedia of Genes and Genomes (KEGG) analysis showed that these DEGs were mainly associated with metabolic pathways, the peroxisome proliferator-activated receptor (PPAR) signaling pathway, regulation of lipolysis in adipocytes, the tumor necrosis factor (TNF) signaling pathway, and the FoxO signaling pathway. The 30 most closely related genes among the DEGs were identified from the protein–protein interaction (PPI) network and verified by real-time quantification during 3T3-L1 preadipocyte differentiation. In conclusion, we obtained a list of consistent DEGs during adipogenesis through integrated analysis, which may offer potential targets for the regulation of adipogenesis and treatment of adipose dysfunction.

## 1. Introduction

When preadipocytes differentiate into mature adipocytes during adipogenesis, adipose tissue gains its specific physiological functions: being the primary storage site for fatty acids and the largest endocrine organ. Much of our understanding of adipogenesis comes from in vitro studies of preadipocyte models (e.g., the 3T3-L1 and 3T3-F442A cell lines) [1,2]. Adipogenesis (adipocyte differentiation) is a multistep biological process that is highly controlled. The expression of several transcription factors and adipogenic genes during adipogenesis causes adipocyte development. During the terminal differentiation stage, the cell morphology changes dramatically (from fibroblastic to spherical), and preadipocytes synchronously gain the characteristics of mature adipocytes. Glucose transporters, enzymes involved in triglyceride metabolism, insulin receptors, and adipocyte-secreted products all show increased activity and quantity, which is necessary for lipid metabolic balance and the inflammatory response. In the past few decades, a number of regulatory factors related to adipogenic programs have been gradually unearthed.

Some studies determined the gene expression profiles during adipogenesis, and a large number of differentially expressed genes (DEGs) related to the initiation of adipogenesis have been identified [3,4,5,6,7]. However, a frequent cause of confusion is the inconsistency of these results due to differences in sample batch, detection platforms, and data processing methods. Therefore, each independent experiment has certain limitations. We need to integrate these results to find credible DEGs that are stable in multiple independent studies using an unbiased approach. In this way, we can fully utilize multiple expression profiles obtained by high-throughput technology to find reliable and effective molecular targets. In this study, to identify DEGs associated with adipogenesis, the robust rank aggregation (RRA) approach was used to integrate multiple ranked gene lists [8]. The RRA method is a powerful ordering algorithm, and for all of the genes in the final ranked gene list, this method assigns a *p*-value to estimate significance probabilities indicating that the result is better than expected by chance [9]. The size of the *p*-value corresponds to the position and significance of the corresponding gene in the final ranked list, meaning that the smaller the *p*-value is, the higher the ranking position of the gene.

In this study, to more accurately identify DEGs associated with adipogenesis, we reanalyzed five expression profile datasets from a public expression database and then integrated these results. With these DEGs, a number of subsequent bioinformatic analyses were performed. Here, we aimed to explore the main pathways and processes associated with adipocyte differentiation and provide key targets for regulating adipose dysfunction and metabolic disorders.

## 2. Results

### 2.1. Expression Profile Datasets and Identification of Differentially Expressed Genes (DEGs) Associated with Adipogenesis

**Figure 1:**
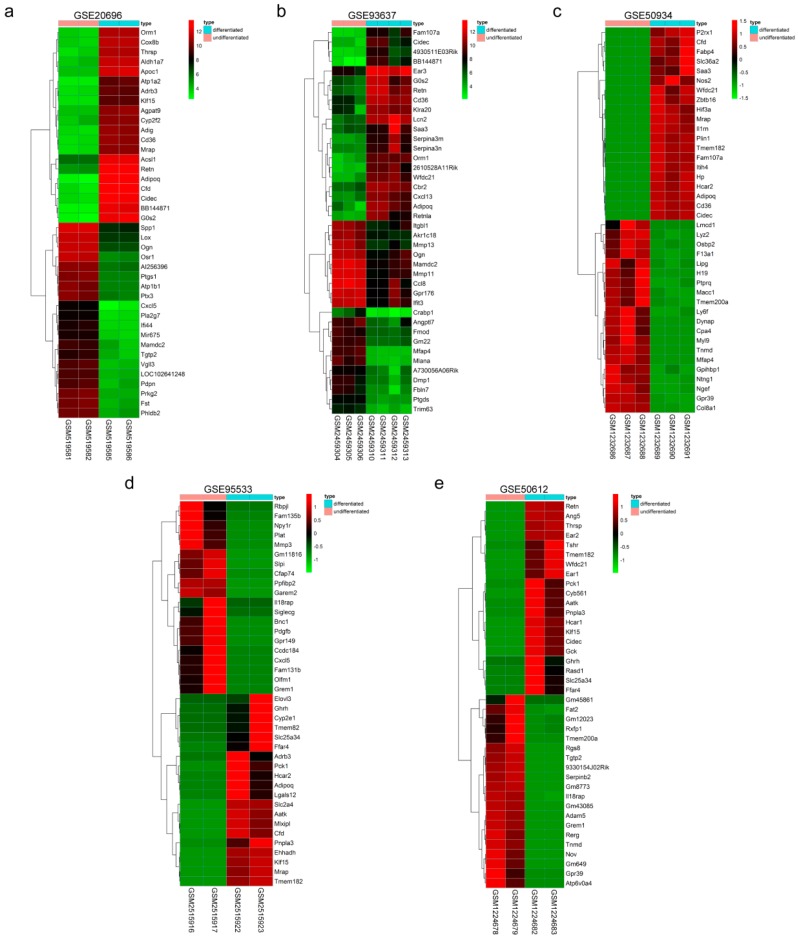
Hierarchical clustering heatmap of the top 20 up- and down-regulated differentially expressed genes (DEGs) screened in each Gene Expression Omnibus (GEO) dataset. (**a**) GSE20696 data, (**b**) GSE93637 data, (**c**) GSE50934 data, (**d**) GSE95533 data, and (**e**) GSE50612 data. Each row represents one gene, and each column represents one sample. Red indicates that the expression of genes is relatively upregulated, and green indicates that the expression of genes is relatively downregulated.

Five expression profile datasets from the Gene Expression Omnibus (GEO) database were selected. In total, 24 samples were obtained, including 12 undifferentiated 3T3-L1 cell samples and 12 differentiated 3T3-L1 cell samples (Table 1). The GSE20696 [7] and GSE93637 [3] expression microarray datasets were standardized, and DEGs were screened with the limma package (|Log_2_ fold change (log_2_FC)| > 1, and corrected *p*-value < 0.05). The high-throughput sequencing datasets GSE50934 [6], GSE95533 [4] and GSE50612 [5] were mapped, and the expression level was calculated. DEGs were screened by the DESeq2 package (|log_2_FC| > 1 and corrected *p*-value < 0.05). The numbers of DEGs in these datasets are shown in Table 1. The cluster heatmaps of the top 40 DEGs from the two sets of sample data included in each of the five expression profile datasets are shown in Figure 1a–e.

### 2.2. Identification of DEGs Associated with Adipogenesis Using Integrated Bioinformatics

**Figure 2:**
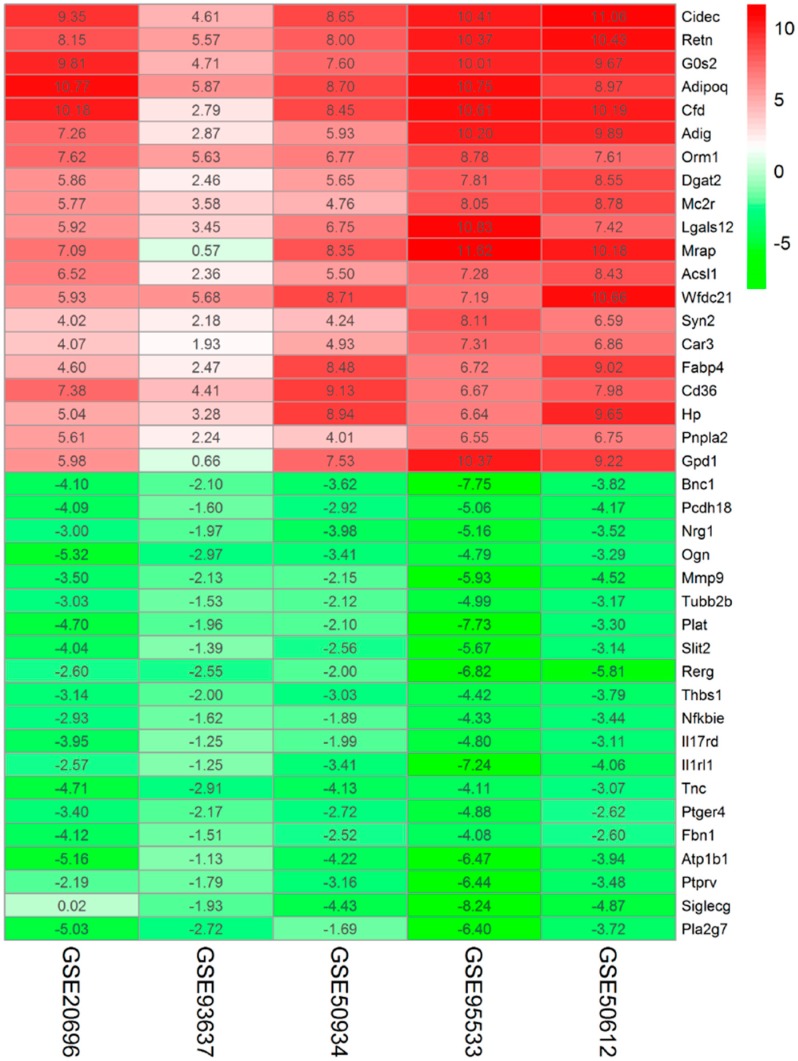
Heatmap of the top 20 up- and down-regulated DEGs in the integrated analysis. Each row represents one gene, and each column represents one dataset. Red indicates that the expression of genes is relatively upregulated, and green indicates that the expression of genes is relatively downregulated. The number in each rectangle represents the value of log_2_FC.

After the above five adipogenesis datasets were reanalyzed by the limma [10] or DESeq2 [11] package, five gene lists ranked according to log_2_FC value were obtained and then analyzed by RRA (|log_2_FC| > 1 and corrected *p*-value < 0.05). Through rank analysis, we identified 386 DEGs, with 230 upregulated genes and 156 downregulated genes. A heatmap of the top 20 up- and down-regulated genes is shown in Figure 2. The integrated analysis results for the top 20 up- and down-regulated DEGs are shown in Table 2.

### 2.3. Enrichment Analysis of the Gene Ontology (GO) Terms of the DEGs

**Figure 3:**
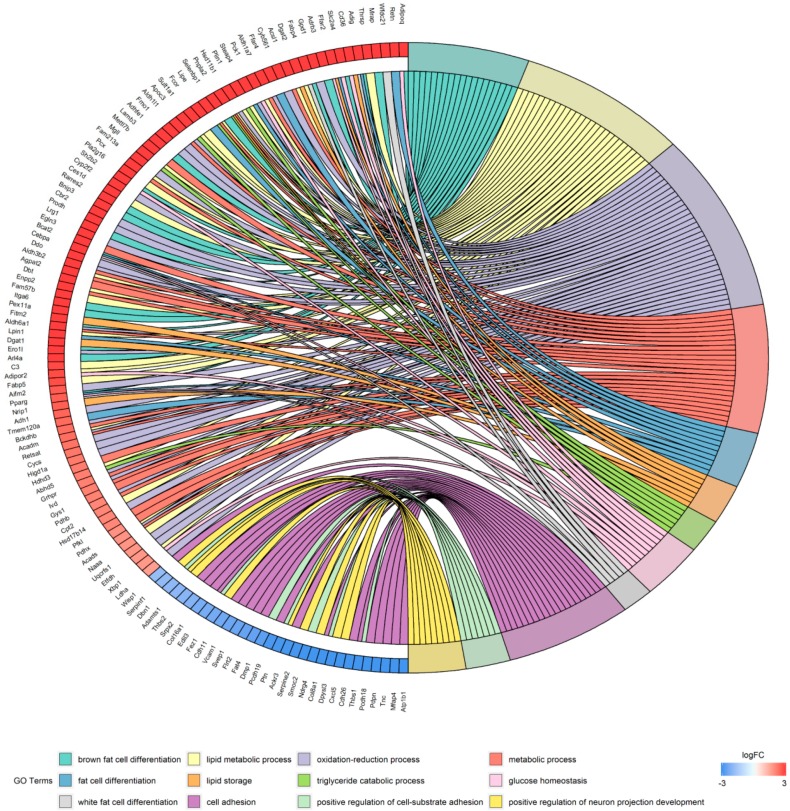
Distribution of genes that are up- and down-regulated during adipogenesis for different GO biological processes.

Significant results from the Gene Ontology (GO) term analysis of DEGs associated with adipogenesis are shown in Table 3. The enrichment results for the biological process (BP) GO category showed that the upregulated genes were primarily involved in fat cell differentiation, lipid metabolic processes, and oxidation-reduction processes. The downregulated genes were primarily concentrated in cell adhesion, positive regulation of cell-substrate adhesion, and positive regulation of neuron projection development. The enrichment results for cell composition (CC) GO category showed that the upregulated genes were primarily concentrated in mitochondria, lipid particles, and the cytosol. The downregulated genes were mainly enriched in extracellular regions, the proteinaceous extracellular matrix, and the extracellular matrix. The enrichment results for the molecular function (MF) GO category showed that the upregulated genes were mainly enriched in oxidoreductase activity. The downregulated genes were mainly enriched in heparin binding, calcium ion binding, and integrin binding. Moreover, the correspondence between genes and biological processes is shown in Figure 3.

### 2.4. Kyoto Encyclopedia of Genes and Genomes (KEGG) Pathway Analysis of DEGs

**Figure 4:**
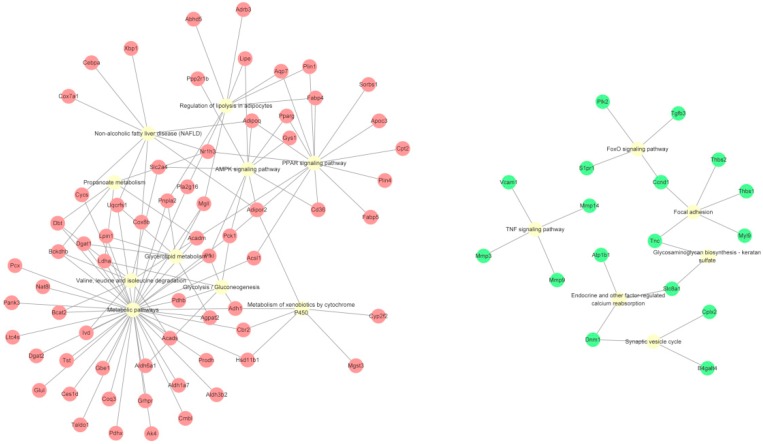
Distribution of up- and down-regulated genes associated with adipogenesis in different KEGG pathways. The upregulated genes (red), downregulated genes (green), and pathway nodes (yellow) are represented by circles.

The top 20 significant pathways for the upregulated genes were selected (Table 4). The upregulated genes were mainly enriched in metabolic pathways, the proliferator-activated receptor (PPAR) signaling pathway, and regulation of lipolysis in adipocytes, and were also enriched in some adipogenesis-related pathways, including the AMP-activated protein kinase (AMPK) signaling pathway, fat digestion and absorption, adipocytokine signaling pathway, and insulin signaling pathway. On the other hand, 11 significant pathways for the downregulated genes were filtered out (Table 4). Except for some diseases and cancer-related pathways, the downregulated genes were mainly enriched in the tumor necrosis factor (TNF) signaling pathway, forkhead box O (FoxO) signaling pathway, and some fundamental biochemical processes (such as focal adhesion, endocrine and other factor-regulated calcium reabsorption, and glycosaminoglycan biosynthesis—keratan sulfate). Cytoscape was used to visualize the relationship between genes and pathways. The genes and pathway nodes are represented by circles. The results are shown in Figure 4.

### 2.5. Protein–Protein Interaction (PPI) Network Construction and Module Analysis of DEGs

**Figure 5:**
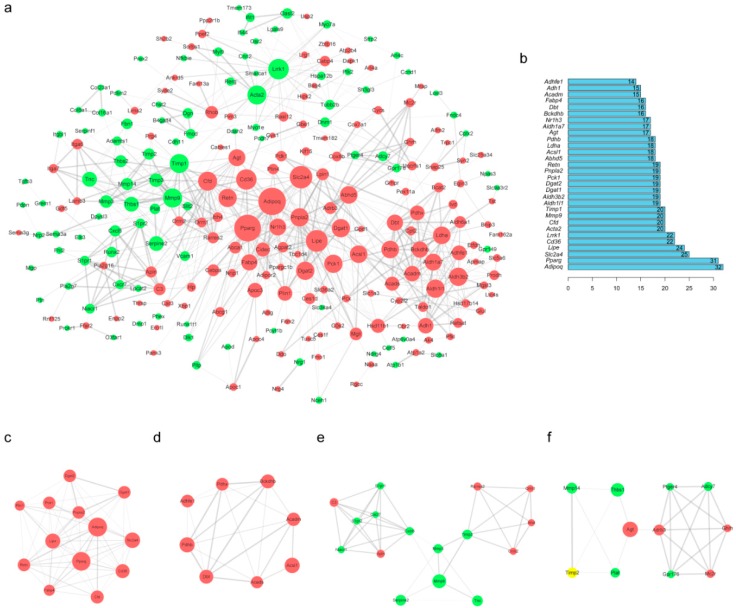
Protein–protein interaction (PPI) network construction and module analysis of DEGs associated with adipogenesis. (**a**) Using Cytoscape software, the PPI network was visualized (isolated nodes were removed). The node size represents the node degree (a larger size indicates a higher degree). The width and transparency of the edge indicate the combined score of the edge (a wider or more opaque edge indicates a higher combined score). (**b**) Top 30 genes with the highest degrees in the PPI network. (**c**–**f**) Molecular Complex Detection (MCODE) module screening for the DEGs, including module 1 (score = 11), module 2 (score = 6), module 3 (score = 5.467), and module 4 (score = 4.6).

To analyze the interaction among DEG expression products, the Search Tool for the Retrieval of Interacting Genes/Proteins (STRING) database [12] was used to construct a PPI network. A total of 247 nodes and 751 edges were obtained with a combined score >0.4, as shown in Figure 5a (isolated nodes were ignored). The top 30 highest degree nodes are shown in Figure 5b. Among these genes, *adiponectin*, *C1Q and collagen domain containing* (*Adipoq)* showed the highest node degree, which was 32. Then, nine modules were selected using the plug-in Molecular Complex Detection (MCODE) to screen the above PPI network. The top 4 modules are shown in Figure 5c–f. In addition, functional annotations for these top 30 genes (only *leucine-rich repeat kinase 1* (*Lrrk1)*, *actin*, *alpha 2*, *smooth muscle*, *aorta (Acta2)*, *matrix metallopeptidase 9 (Mmp9)*, and *tissue inhibitor of metalloproteinase 1* (*Timp1)* were downregulated) were implemented (Table 5). GO analysis showed that the genes were mainly related to lipid metabolism, oxidation-reduction, and fat cell differentiation. KEGG analysis showed that they were mainly associated with the PPAR signaling pathway, metabolism pathways, and the AMPK signaling pathway.

### 2.6. Verification of Changes in DEG Expression During 3T3-L1 Preadipocyte Differentiation

**Figure 6:**
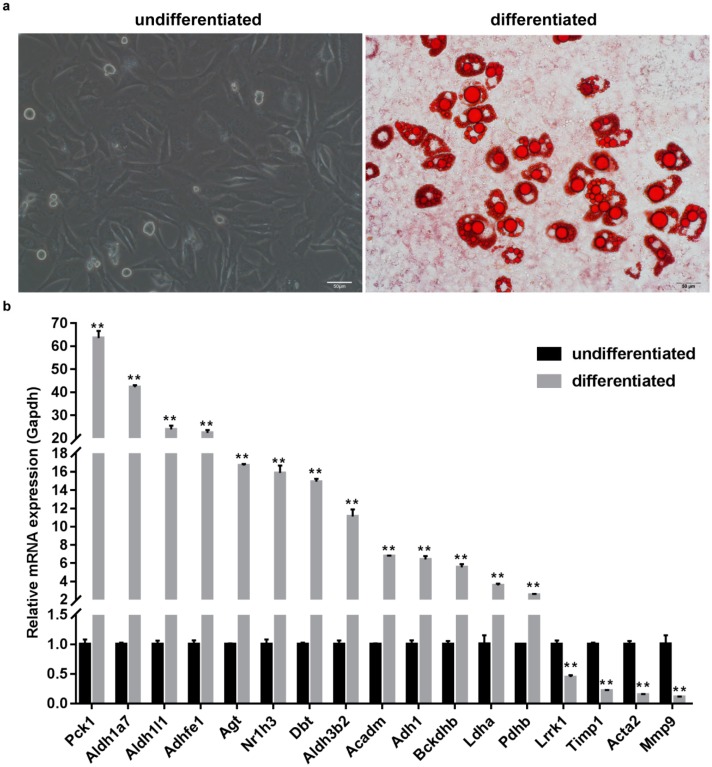
Verification of differential expression of key genes during 3T3-L1 preadipocyte differentiation. (**a**) Undifferentiated (Day 0) and differentiated (Day 8, Oil red O staining) 3T3-L1 cells under the induced differentiation process. (**b**) Relative expression levels of potential key genes were measured in undifferentiated and differentiated 3T3-L1 cells by qRT-PCR. Three independent experiments were performed for all of these groups, and the quantitative results are expressed as the mean ± SD of three independent experiments. ** indicates *p* < 0.01.

We believe that the top 30 highest degree genes in the PPI network may play a key regulatory role in the process of adipogenic differentiation. Here, we measured their expression levels before and after 3T3-L1 preadipocyte differentiation. First, we induced differentiation of 3T3-L1 preadipocytes and performed oil red O staining (Figure 6a). The results showed that the lipid droplets were numerous and large after differentiation. Next, we determined the expression levels of 17 potential key genes in undifferentiated and differentiated 3T3-L1 cells through quantitative real-time PCR (qRT-PCR) (Figure 6b). The results showed that their expression varied significantly during adipogenesis. The direction of change was consistent with the results of the previous integrated bioinformatic analysis.

## 3. Discussions

Adipose tissue is composed of many cell types, and mature adipocytes account for only two-thirds of adipose tissue. Undifferentiated cells are also found in adipose tissue, including preadipocytes and stem cells. Stem cells have the potential to differentiate into various types of cells, and the direction of the differentiation of preadipocytes has been determined. Proliferation and differentiation of preadipocytes are essential for the continued development and maintenance of adipose tissue. Spalding et al. found that almost 50% of human subcutaneous fat is renewed every 8 years, suggesting that adipocytes are a dynamic cell type that undergoes constant substitution by newborn adipocytes [13]. In other words, adipogenesis fundamentally determines the expansion and functional properties of adipose tissue. Considering the occurrence of increases in public health problems caused by adipose dysfunction and metabolic disorders (such as obesity, diabetes mellitus, insulin resistance, and cardiovascular disease) on a global scale, it is necessary to explore the fundamental molecular mechanism of adipogenesis [14].

With the development of high-throughput technology, a large amount of transcriptome data is generated and uploaded to a public expression database. Fully exploiting these large datasets can provide good value to life science research. Given the inevitable errors among independent experiments, we urgently need to integrate the results of various experiments to more accurately identify the intrinsic components and elucidate the major molecular mechanisms. In this study, we integrated five gene expression profile datasets of the adipogenesis processes from different independent experiments. A total of 386 DEGs were identified, including 230 upregulated genes and 156 downregulated genes. The upregulated gene list contained many fat marker genes (such as *Adipoq*, *peroxisome proliferator activated receptor gamma* (*Pparg*), and *solute carrier family 2 (facilitated glucose transporter), member 4* (*Slc2a4*)), and a large number of adipogenic differentiation studies have been carried out around them. The downregulated gene list includes the well-known antiadipogenesis gene *delta-like 1* (*Dlk1*), but many of the other genes related to adipogenic differentiation have rarely been reported. In general, the role of these genes with dramatic changes in expression during adipogenesis deserves a deeper understanding. This study provides a reliable collection of DEGs associated with adipogenic processes, providing a large number of potential subjects for subsequent research.

In addition, functional annotation of DEGs was performed to fully understand the processes and pathways in which they participate. GO analysis of the upregulated genes revealed that adipocyte differentiation and concomitant activities, such as lipid metabolism and redox activity, were major biological processes in which they were involved. The KEGG analysis of upregulated genes showed consistent results, with the PPAR signaling pathways and lipid metabolism-related pathways (such as metabolic pathways and regulation of lipolysis in adipocytes) playing a dominant role. The PPAR signaling pathway has been extensively studied as a core pathway in the process of adipogenic differentiation. Peroxisome proliferator-activated receptor gamma (PPARG, encoded by *Pparg*) is the master regulator of adipose differentiation; its expression is necessary for initiating differentiation and maintaining a differentiated state, and a large number of prodifferentiation factors function through its activation [15]. The GO and KEGG analyses of downregulated genes also showed consistent results, with biological processes and pathways involved in adhesion and activity of the extracellular matrix (ECM) predominating. Phenotypic changes in cells during fat differentiation were apparent and were accompanied by changes in the levels and type of ECM components [16], and proteolytic degradation of preadipocytes by ECM components is required for cell-shape changes, adipocyte-specific gene expression, and lipid accumulation [17]. On the other hand, downregulated genes are also enriched in some antiadipogenic differentiation pathways, such as the TNF signaling pathway [18] and the FoxO signaling pathway [19].

A PPI network of DEG-encoded proteins was constructed and the 30 most closely related genes were selected. Further functional annotation of these key genes was performed, and the results were consistent with the functional annotations of the entire set of DEGs. They were mainly concentrated in the same biological processes and pathways involved in adipogenesis. The findings further suggested that the 30 key genes that we screened were credible because the 30 key genes were sufficient to represent all the DEGs and reflect the main activities of adipogenesis. We believe that these genes with the highest node degree play a major regulatory role in multiple processes of adipogenic differentiation. Some of these genes are well-known as key genes in fat differentiation and lipid metabolism functions, such as the transcriptional regulation of adipogenesis (*Pparg*), insulin sensitive glucose transport (*Slc2a4*) [20], fatty acid transport (*fatty acid binding protein 4* (*Fabp4*), *CD36 Molecule* (*Cd36*)) [21,22], triacylglycerol (TAG) synthesis (*diacylglycerol O-acyltransferase 1* (*Dgat1*), *diacylglycerol O-acyltransferase 1* (*Dgat2*), *acyl-CoA synthetase long-chain family member 1* (*Acsl1*)) [23,24,25,26], lipolysis and its regulation (*lipase, hormone sensitive* (*Lipe*), *patatin-like phospholipase domain containing 2* (*Pnpla2*), *abhydrolase domain containing 5* (*Abhd5*)) [27,28], and the endocrine functions of adipocytes (*Adipoq*, *complement factor D* (*Cfd*), *resistin* (*Retn*)) [29,30]. In addition, tissue inhibitor of metalloproteinases-1 (TIMP1, encoded by *Timp1*) is a natural inhibitor of matrix metalloproteinases (MMPs) [31], a cluster of peptidases that are involved in degrading and remodeling the ECM [32,33]. MMP9 (a member of the MMP family, encoded by *Mmp9*) is secreted by adipocytes, and its proteolytic activity was induced during adipocyte differentiation [34]. The *Nr1h3* gene encodes a nuclear receptor, Nuclear Receptor Subfamily 1 Group H Member 3 (NR1H3, also known as LXRα), which is a vital regulator in hepatic de novo lipogenesis and lipid homeostasis [35]. NR1H3 exhibits ligand-dependent activation activity and is activated primarily by cellular cholesterols, leading to the induction of transcription of a downstream nuclear transcriptional factor, sterol regulatory element binding transcription factor 1 (SREBF1) [36]. The *Pck1* gene is mainly responsible for the regulation of gluconeogenesis [37] and is closely related to diabetes mellitus and obesity [38]. Cytosolic isozyme of phosphoenolpyruvate carboxykinase (PEPCK-C, encoded by *Pck1*) stimulates the transformation of oxaloacetate to phosphoenolpyruvate (PEP) in fat cells [39]. As a member of the aldehyde dehydrogenase 3 (ALDH3) family, aldehyde dehydrogenase 3 family member B2 (ALDH3B2, encoded by *Aldh3b2*) is localized in lipid droplets and is responsible for the removal of lipid aldehydes, exhibiting broad substrate specificity towards medium- and long-chain aldehydes [40]. The remaining genes with high node degrees, such as *Lrrk1*, *aldehyde dehydrogenase 1 family, member L1* (*Aldh1l1*)*,* and *alcohol dehydrogenase, iron containing, 1* (*Adhfe1*), are very rarely reported to be related to adipogenesis. These genes deserve more attention from frontline researchers, and their potential role in adipogenesis requires further experimental verification.

Finally, we verified the expression changes in some key genes before and after 3T3-L1 preadipocyte differentiation. Combining the results of integrated bioinformatic analyses with the results of gene expression measurements, this study has greatly narrowed the range of potential key genes and provides high-value targets for subsequent adipogenic differentiation research.

## 4. Materials and Methods

### 4.1. Gene Expression Profile Data

The gene expression profile datasets GSE50934, GSE95533, GSE50612, GSE93637, and GSE20696 were downloaded from the GEO database (https://www.ncbi.nlm.nih.gov/geo/). The platforms of the five GEO datasets were GPL13112 Illumina HiSeq 2000 (Mus musculus) for the GSE50934 and GSE50612 datasets, GPL18480 Illumina HiSeq 1500 (Mus musculus) for the GSE95533 dataset, and GPL1261 [Mouse430_2] Affymetrix Mouse Genome 430 2.0 Array for the GSE93637 and GSE20696 datasets.

### 4.2. Identification of DEGs

For the raw data of GSE50934, GSE95533, and GSE50612, reads were mapped to the mouse genome (GRCm38) using HiSat2 [41], and annotated genes were quantified with featureCounts [42]. Differential expression analysis was performed using the DESeq2 R package. For the raw data of GSE93637 and GSE20696, normalization and differential expression analysis were performed using the limma R package. DESeq2 and limma R package were all from the Bioconductor project (https://www.bioconductor.org/). All the R packages used in this study were deployed in the programming language R (version 3.3.3, Auckland, New Zealand).

### 4.3. Integration of Gene Expression Profile Data

The RRA method was based on the assumption that all genes are unordered in each list. In this study, only the overlapping DEGs were used for the integrated analysis, and the five lists of genes were ranked by expression level in the five datasets. The RobustRankAggreg package of programming language R was downloaded from the Comprehensive R Network (https://cran.r-project.org/).

### 4.4. GO Term and KEGG Pathway Enrichment Analyses

For functional annotation of the DEGs, GO term enrichment analysis was performed using the online Visualization and Integrated Discovery (DAVID) software (https://david.ncifcrf.gov/) [43,44], for the three GO categories (BP, CC and MF). In addition, KEGG pathway enrichment analysis was carried out using KEGG Orthology-Based Annotation System (KOBAS) software (version 3.0, Peking, China (http://kobas.cbi.pku.edu.cn/)) [45,46]. Significant enrichment was considered for a corrected *p*-value (FDR) < 0.05.

### 4.5. PPI Network Construction and Module Analysis

The list of genes was mapped to the STRING database (http://www.string-db.org/) to construct a functional protein association network. Then, the PPI network was visualized with Cytoscape software (version 3.6.0, Washington, DC, USA) (http://www.cytoscape.org/). The degree of a node is the number of edges (interactions) incident to that node. A node is important if it links to many other nodes. The genes at the top of the degree distribution (≥90% percentile) in the network were defined as key genes (central genes). The plug-in MCODE (http://apps.cytoscape.org/apps/mcode) was used to scan the PPI network to identify densely connected regions. A *p*-value < 0.05 was considered statistically significant.

### 4.6. Cell Culture, Differentiation, and Lipid Droplet Staining

The culture and differentiation of 3T3-L1 preadipocytes were performed as previously described in Reference [47]. For lipid droplet (LD) staining, cells were washed with phosphate buffer saline (PBS) three times and then fixed in 4% paraformaldehyde for 30 min at room temperature. After washing three times with PBS, the cells were stained with a 60% filtered oil red O (Sigma, St. Louis, MO, USA) stock solution (0.3 g/100 mL of isopropanol). The cells were then rinsed three times with PBS. All images were obtained using an inverted fluorescence microscope FV500-IX71 (Olympus, Tokyo, Japan).

### 4.7. qRT-PCR Validation of Key Genes

Total RNA from undifferentiated and differentiated 3T3 cells was extracted using RNAiso (Takara, Dalian, China). A PrimeScriptTM RT Reagent Kit with gDNA Eraser (Takara) was used to perform reverse transcription of total RNA. A SYBR^®^ Premix Ex TaqTM ΙΙ Kit (Takara) was used to carry out qRT-PCR with an ABI 7500 Real-Time PCR system (Applied Biosystems, Foster, CA, USA). Gapdh served as an internal reference to normalize gene expression levels via the 2^−∆∆*C*t^ method, as in Reference [48]. Some of the key genes whose expression trends during the adipogenesis of 3T3-L1 preadipocytes were known were excluded, and qRT-PCR validation was performed for the remaining genes.

### 4.8. Statistical Analysis

All the quantitative experiments were performed three times, and the quantitative results were expressed as the mean ± SD. Comparisons between two sets of groups were analyzed using the Student’s two-tailed *t*-test with GraphPad Prism 7. Statistical significance depended on a value of *p* < 0.05 (significant) or *p* < 0.01 (extremely significant).

## 5. Conclusions

Given the limitations of independent experiments, analyzing different datasets often yields different results, especially when performing differential expression analysis. In this study, we used the RRA method to integrate the results of five high-throughput datasets well, and we obtained consistent DEGs showing an association with adipogenesis. This work will contribute to the study of adipogenesis in the future, thus promoting our understanding of the molecular mechanisms underlying adipogenesis, and it may offer potential targets for the regulation of adipogenesis and the treatment of adipose dysfunction. Further experimental verification is needed to explore the specific functions of these DEGs.

## Figures and Tables

**Table 1:** Undifferentiated and differentiated 3T3-L1 cell samples from the NCBI Gene Expression Omnibus (GEO) database used in this study.

Reference	GEO	Platform	Study Type	Differentiation Stage of Samples		Number of DEGs (Up/Down)
				Undifferentiated	Differentiated	
Mikkelsen, T.S. et al. (2010) [7]	GSE20696	GPL1261	microarray	GSM519581 GSM519582	GSM519585 GSM519586	2898 (1425/1473)
Romero, M. et al. (2018) [3]	GSE93637	GPL1261	microarray	GSM2459304 GSM2459305 GSM2459306	GSM2459310 GSM2459311 GSM2459312 GSM2459313	1119 (591/528)
Duteil, D. et al. (2014) [6]	GSE50934	GPL13112	RNA-seq	GSM1232686 GSM1232687 GSM1232688	GSM1232689 GSM1232690 GSM1232691	2685 (1552/1133)
Siersbæk, R. et al. (2017) [4]	GSE95533	GPL18480	RNA-seq	GSM2515916 GSM2515917	GSM2515922 GSM2515923	4416 (2197/2219)
Al, A.H. et al. (2015) [5]	GSE50612	GPL13112	RNA-seq	GSM1224678 GSM1224679	GSM1224682 GSM1224683	3966 (2170/1796)

**Table 2:** Top 20 up- and down-regulated DEGs identified during adipogenesis via integrated analysis.

Top 20 Upregulated Genes				Top 20 Downregulated Genes			
Symbol	log_2_FC	*p*-Value	Corrected *p*-Value	Symbol	log_2_FC	*p*-Value	Corrected *p*-Value
*Cidec*	8.815805	2.15 × 10^−15^	5.34 × 10^−11^	*Bnc1*	−4.27787	7.38 × 10^−12^	1.83 × 10^−7^
*Retn*	8.50511	5.14 × 10^−15^	1.28 × 10^−10^	*Pcdh18*	−3.56837	8.39 × 10^−11^	2.09 × 10^−6^
*G0s2*	8.359012	2.39 × 10^−14^	5.94 × 10^−10^	*Nrg1*	−3.52592	1.30 × 10^−10^	3.24 × 10^−6^
*Adipoq*	9.012189	1.81 × 10^−13^	4.51 × 10^−9^	*Ogn*	−3.95531	2.77 × 10^−10^	6.89 × 10^−6^
*Cfd*	8.441624	1.81 × 10^−13^	4.51 × 10^−9^	*Mmp9*	−3.64602	3.46 × 10^−10^	8.60 × 10^−6^
*Adig*	7.229626	1.17 × 10^−12^	2.90 × 10^−8^	*Tubb2b*	−2.96698	3.77 × 10^−10^	9.37 × 10^−6^
*Orm1*	7.280763	9.69 × 10^−12^	2.41 × 10^−7^	*Plat*	−3.95792	4.36 × 10^−10^	1.09 × 10^−5^
*Dgat2*	6.066281	1.06 × 10^−11^	2.63 × 10^−7^	*Slit2*	−3.3568	7.55 × 10^−10^	1.88 × 10^−5^
*Mc2r*	6.186546	1.10 × 10^−11^	2.75 × 10^−7^	*Rerg*	−3.95606	1.10 × 10^−9^	2.73 × 10^−5^
*Lgals12*	6.873706	1.67 × 10^−11^	4.15 × 10^−7^	*Thbs1*	−3.27764	1.16 × 10^−9^	2.87 × 10^−5^
*Mrap*	7.563215	2.17 × 10^−11^	5.39 × 10^−7^	*Nfkbie*	−2.84309	1.82 × 10^−9^	4.53 × 10^−5^
*Acsl1*	6.016773	3.73 × 10^−11^	9.29 × 10^−7^	*Il17rd*	−3.02351	2.32 × 10^−9^	5.77 × 10^−5^
*Wfdc21*	7.632637	4.86 × 10^−11^	1.21 × 10^−6^	*Il1rl1*	−3.70607	2.50 × 10^−9^	6.21 × 10^−5^
*Syn2*	5.030408	8.88 × 10^−11^	2.21 × 10^−6^	*Tnc*	−3.78602	4.22 × 10^−9^	1.05 × 10^−4^
*Car3*	5.021359	1.14 × 10^−10^	2.83 × 10^−6^	*Ptger4*	−3.15885	4.22 × 10^−9^	1.05 × 10^−4^
*Fabp4*	6.257684	1.34 × 10^−10^	3.32 × 10^−6^	*Fbn1*	−2.96534	4.99 × 10^−9^	1.24 × 10^−4^
*Cd36*	7.115236	1.48 × 10^−10^	3.69 × 10^−6^	*Atp1b1*	−4.18269	5.09 × 10^−9^	1.27 × 10^−4^
*Hp*	6.709448	1.60 × 10^−10^	3.98 × 10^−6^	*Ptprv*	−3.41098	6.87 × 10^−9^	1.71 × 10^−4^
*Pnpla2*	5.031089	1.77 × 10^−10^	4.40 × 10^−6^	*Siglecg*	−3.88948	7.62 × 10^−9^	1.90 × 10^−4^
*Gpd1*	6.752793	2.03 × 10^−10^	5.05 × 10^−6^	*Pla2g7*	−3.91415	7.64 × 10^−9^	1.90 × 10^−4^

**Table 3:** Gene Ontology (GO) term enrichment analysis of DEGs associated with adipogenesis.

DEGs	Term	Category	Count	FDR
up	brown fat cell differentiation	BP	19	1.63 × 10^−23^
	lipid metabolic process	BP	27	3.04 × 10^−8^
	oxidation-reduction process	BP	32	6.77 × 10^−8^
	metabolic process	BP	23	3.03 × 10^−5^
	fat cell differentiation	BP	10	4.76 × 10^−4^
	lipid storage	BP	7	8.27 × 10^−4^
	triglyceride catabolic process	BP	6	4.12 × 10^−3^
	glucose homeostasis	BP	10	3.70 × 10^−2^
	white fat cell differentiation	BP	5	4.44 × 10^−2^
	mitochondrion	CC	52	9.65 × 10^−8^
	lipid particle	CC	12	2.11 × 10^−7^
	cytosol	CC	42	8.61 × 10^−3^
	intracellular membrane-bounded organelle	CC	24	1.60 × 10^−2^
	mitochondrial membrane	CC	9	2.25 × 10^−2^
	oxidoreductase activity	MF	27	3.98 × 10^−6^
down	cell adhesion	BP	20	8.40 × 10^−6^
	positive regulation of cell-substrate adhesion	BP	7	1.19 × 10^−3^
	positive regulation of neuron projection development	BP	9	1.74 × 10^−2^
	extracellular region	CC	51	4.64 × 10^−14^
	proteinaceous extracellular matrix	CC	24	2.78 × 10^−13^
	extracellular matrix	CC	21	1.28 × 10^−10^
	extracellular space	CC	41	1.82 × 10^−9^
	basement membrane	CC	11	4.33 × 10^−6^
	calcium ion binding	MF	24	4.46 × 10^−6^
	integrin binding	MF	9	1.34 × 10^−3^
	heparin binding	MF	13	2.56 × 10^−6^

**Table 4:** Kyoto Encyclopedia of Genes and Genomes (KEGG) pathway enrichment analysis of DEGs in adipogenesis.

DEGs	Term	Count	FDR
up	Metabolic pathways	42	1.34 × 10^−16^
	PPAR signaling pathway	15	3.33 × 10^−15^
	Regulation of lipolysis in adipocytes	9	1.20 × 10^−8^
	AMPK signaling pathway	10	5.06 × 10^−7^
	Valine, leucine and isoleucine degradation	7	3.31 × 10^−6^
	Non-alcoholic fatty liver disease (NAFLD)	9	2.05 × 10^−5^
	Propanoate metabolism	5	5.31 × 10^−5^
	Glycerolipid metabolism	6	5.31 × 10^−5^
	Metabolism of xenobiotics by cytochrome P450	6	7.77 × 10^−5^
	Glycolysis/Gluconeogenesis	6	7.77 × 10^−5^
	Pyruvate metabolism	5	8.57 × 10^−5^
	Nitrogen metabolism	4	8.57 × 10^−5^
	Insulin resistance	7	8.57 × 10^−5^
	Fat digestion and absorption	5	8.57 × 10^−5^
	Adipocytokine signaling pathway	6	8.90 × 10^−5^
	Carbon metabolism	7	1.09 × 10^−4^
	Proximal tubule bicarbonate reclamation	4	1.63 × 10^−4^
	Fatty acid degradation	5	1.65 × 10^−4^
	Glucagon signaling pathway	6	4.20 × 10^−4^
	Biosynthesis of amino acids	5	1.26 × 10^−3^
down	Malaria	4	7.99 × 10^−3^
	Transcriptional misregulation in cancer	6	7.99 × 10^−3^
	Rheumatoid arthritis	4	2.24 × 10^−2^
	Bladder cancer	3	2.67 × 10^−2^
	TNF signaling pathway	4	3.24 × 10^−2^
	Focal adhesion	5	3.24 × 10^−2^
	Endocrine and other factor-regulated calcium reabsorption	3	3.24 × 10^−2^
	Glycosaminoglycan biosynthesis—keratan sulfate	2	3.51 × 10^−2^
	Synaptic vesicle cycle	3	3.51 × 10^−2^
	FoxO signaling pathway	4	3.51 × 10^−2^
	Bile secretion	3	4.35 × 10^−2^

**Table 5:** Enrichment analysis of the top 30 genes with the highest degrees.

Method	Term	Count	FDR	Genes
GO	metabolic process	10	3.98 × 10^−5^	*Dbt*, *Aldh1l1*, *Acsl1*, *Acadm*, *Bckdhb*, *Aldh3b2*, *Pnpla2*, *Aldh1a7*, *Pdhb*, *Lipe*
	oxidation-reduction process	9	1.12 × 10^−2^	*Ldha*, *Aldh1l1*, *Acadm*, *Adhfe1*, *Adh1*, *Bckdhb*, *Aldh3b2*, *Aldh1a7*, *Pdhb*
	lipid storage	4	1.51 × 10^−2^	*Dgat1*, *Cd36*, *Dgat2*, *Pnpla2*
	brown fat cell differentiation	4	3.07 × 10^−2^	*Slc2a4*, *Pparg*, *Fabp4*, *Adipoq*
	negative regulation of sequestering of triglyceride	3	3.54 × 10^−2^	*Pparg*, *Abhd5*, *Pnpla2*
KEGG	PPAR signaling pathway	8	2.89 × 10^−13^	*Acadm*, *Adipoq*, *Fabp4*, *Cd36*, *Acsl1*, *Pck1*, *Pparg*, *Nr1h3*
	Metabolic pathways	13	1.61 × 10^−10^	*Acadm*, *Adh1*, *Bckdhb*, *Dbt*, *Dgat1*, *Acsl1*, *Ldha*, *Pck1*, *Aldh1a7*, *Pnpla2*, *Dgat2*, *Pdhb*, *Aldh3b2*
	AMPK signaling pathway	6	1.88 × 10^−8^	*Adipoq*, *Cd36*, *Lipe*, *Pck1*, *Pparg*, *Slc2a4*
	Glycolysis/Gluconeogenesis	5	3.90 × 10^−8^	*Adh1*, *Ldha*, *Pck1*, *Pdhb*, *Aldh3b2*
	Adipocytokine signaling pathway	5	5.04 × 10^−8^	*Adipoq*, *Cd36*, *Acsl1*, *Pck1*, *Slc2a4*

## Abbreviations

GEO	Gene expression omnibus
DEGs	Differentially expressed genes
RRA	Robust rank aggregation
GO	Gene ontology
KEGG	Kyoto encyclopedia of genes and genomes
PPIMCODE	Protein–protein interactionMolecular complex detection
